# Location Accuracy of INS/Gravity-Integrated Navigation System on the Basis of Ocean Experiment and Simulation

**DOI:** 10.3390/s17122961

**Published:** 2017-12-20

**Authors:** Hubiao Wang, Lin Wu, Hua Chai, Lifeng Bao, Yong Wang

**Affiliations:** 1 A State Key Laboratory of Geodesy and Earth’s Dynamics, Institute of Geodesy and Geophysics, Chinese Academy of Sciences, Wuhan 430077, China; wanghb@whigg.ac.cn (H.W.); linwu@whigg.ac.cn (L.W.); hchai@whigg.ac.cn (H.C.); baolifeng@whigg.ac.cn (L.B.); 2 Department of Earth Sciences, University of Chinese Academy of Sciences, Beijing 100049, China

**Keywords:** INS/gravity-integrated navigation system, marine gravity anomaly, multi-model adaptive Kalman filtering, matching location accuracy, ocean experiment, simulation

## Abstract

An experiment comparing the location accuracy of gravity matching-aided navigation in the ocean and simulation is very important to evaluate the feasibility and the performance of an INS/gravity-integrated navigation system (IGNS) in underwater navigation. Based on a 1′ × 1′ marine gravity anomaly reference map and multi-model adaptive Kalman filtering algorithm, a matching location experiment of IGNS was conducted using data obtained using marine gravimeter. The location accuracy under actual ocean conditions was 2.83 nautical miles (n miles). Several groups of simulated data of marine gravity anomalies were obtained by establishing normally distributed random error $N(u,\sigma^{2})$ with varying mean u and noise variance $\sigma^{2}$. Thereafter, the matching location of IGNS was simulated. The results show that the changes in u had little effect on the location accuracy. However, an increase in $\sigma^{2}$ resulted in a significant decrease in the location accuracy. A comparison between the actual ocean experiment and the simulation along the same route demonstrated the effectiveness of the proposed simulation method and quantitative analysis results. In addition, given the gravimeter (1–2 mGal accuracy) and the reference map (resolution 1′ × 1′; accuracy 3–8 mGal), location accuracy of IGNS was up to reach ~1.0–3.0 n miles in the South China Sea.

## 1. Introduction

Currently, land-based radio navigation and the global navigation satellite system (GNSS) are the dominant tools for aviation and sailing. Underwater navigation, however, is different from space navigation. Although the marine environment helps to conceal underwater submersibles, it poses difficulties in transmission and navigation for them [1,2,3,4]. With the development of the inertial navigation technology and the underwater acoustic location technology, the latter technology has made great progress in terms of short-period underwater navigation and local area location determination, but its capability to locate hidden underwater submersibles over long distances and periods still fails to meet the current military and civilian needs. At present, the location accuracy of inertial navigation systems (INS) reaches up to 1 nautical mile (n mile)/3 days or even greater. However, the location error of INS increases with time, so the navigation must be readjusted and corrected during long-distance and long-period sailing to realize high-precision location determination. To control the increase in the location error, two or more navigation systems are often combined to form an integrated underwater navigation system [5,6,7].

The gravitational field is one of the inherent physical properties of the earth, and has strong stability and anti-interference capacity. It is unevenly distributed and has a changing topology. When a gravimeter is used to obtain gravitational information at the location of a submarine, no energy is externally emitted. Moreover, the submarine does not need to move to the ocean surface to receive the external signals, so there is effective concealment. From the aspects of passivity, autonomy and feasibility, IGNS has become the preferred method for improving the location accuracy without compromising the concealment of underwater submersibles [8,9,10].

With respect to IGNS, many researchers have conducted numerous studies in the field, for instance, characteristic analyses of the marine gravity field model, methods of real-time marine gravity measurement, preprocessing and upward continuation of gravimeter-measured data, matching region partition, and optimization and fusion of various matching algorithms. Thus, important results have been obtained in the field of IGNS [11,12,13,14,15]. However, these studies have focused on the basic principles and simulation experimental methods. Although simulation can be used to some extent to test a gravity matching-aided navigation system, the external factors considered are simplistic and idealized, and the simulation results are not completely convincing.

The technologies used in IGNS are relatively complicated. Further, many researchers are concerned about how to verify the system function and the algorithm accuracy. Verification and analysis through ocean experiments, in addition to simulations, is very important for the practical application of IGNS. To objectively and appropriately determine the system performance and the actual location determination outcomes, we calculated the location accuracy of such a system, based on the route data obtained using a marine gravimeter during an expedition in the South China Sea and an analysis of the gravity field characteristics in the route area. The simulation observation data obtained using a marine gravimeter under different noise conditions along the route were used to analyze the location accuracy. By comparing the results of the ocean experiment and the simulation, we analyzed the effects of the gravimeter accuracy and the error in the gravity anomaly reference map on the location accuracy. In addition, we addressed the location accuracy of IGNS that could be attained in actual ocean conditions, given the precision of the current gravimeters and the resolution and accuracy of the reference maps.

## 2. Principles of IGNS and Variation Characteristics of Gravity Anomaly

### 2.1. Principles of IGNS

For IGNS, the variation characteristics of an information database composed of high-resolution gravity reference maps were used to acquire the carrier location information [16,17]. Gravity matching-aided navigation primarily uses a variety of methods to compare the gravity values obtained using marine gravimeters and those stored in reference maps, thereby determining the optimal matching point according to the degree of fit between the two types of gravity values. Algorithms of gravity matching-aided navigation can largely be divided into two types, i.e., related matching algorithms represented by terrain contour matching (TERCOM) [18,19] and interactive closest contour point (ICCP) [20], and multi-model Kalman filtering algorithms represented by the Sandia inertial terrain-aided navigation (SITAN) [21,22]. There are other algorithms, such as neural networks and particle filtering [23,24,25]. Unlike the related matching algorithms, SITAN uses recursive Kalman filtering to calculate the carrier location in real time. It is insensitive to speed or heading errors and allows the carrier to move flexibly. Therefore, the SITAN algorithm was used in the following ocean experiment and simulation.

**Figure 1:**
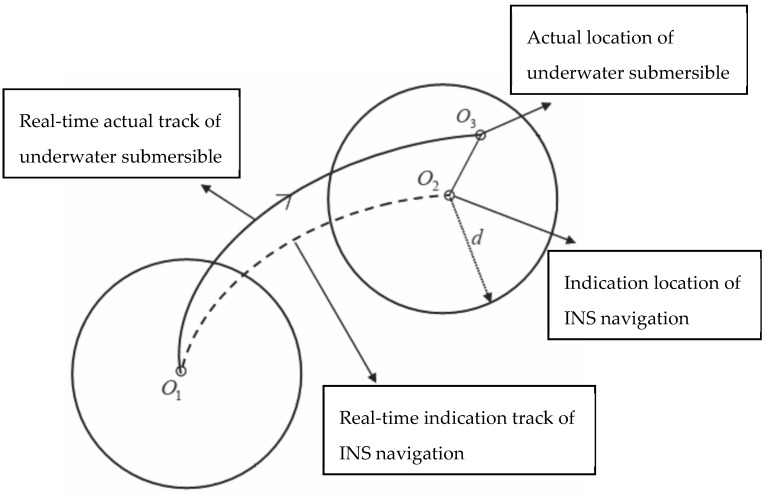
Comparison between actual location of underwater submersible and INS navigation location.

Figure 1 shows the drift of the INS locations with respect to the actual ones. If an underwater submersible sails from $O_{1}$ to $O_{3}$, the INS location at this moment corresponding to the actual location $O_{3}$ is $O_{2}$, because of the accumulated INS location error. The SITAN algorithm for gravity matching-aided navigation has two phases, namely, search and tracking. In the search phase, centered on location $O_{2}$ indicated by the INS, the actual location area having a search radius d of the underwater submersible with 99% confidence was determined on an existing gravity anomaly reference map, based on the INS location accuracy and the circular error probability (CEP). With location $O_{2}$ indicated by the INS as the center of the confidence area, a set of parallel Kalman filters was established in the confidence area to track and calculate the matched locations. These filters were ordered grids that had spacing consistent with the grid resolution of the gravity anomaly reference maps. The corresponding gravity anomaly interpolation was determined from the gravity anomaly reference maps on the basis of the filter distribution. The difference between the gravity anomaly interpolated from a reference map and that observed using a marine gravimeter was considered the measured value, ultimately forming the state equation and the measurement equation (see Equation (1)). The estimated gravity anomaly and location information along the route were obtained using Kalman filtering.

The state equation and the measurement equation of Kalman filtering in IGNS were constructed as follows [26,27]:

(1) $$\{\begin{array}{c} X_{k}=X_{k-1}+W_{k-1}\\ Z_{k}=X_{k}+V_{k} \end{array},$$

If we assume $Z_{k}=g_{meas}-g_{map}$, $Z_{k}$ is the observed value of the measurement equation at time $t_{k}$. Here, $g_{meas}$ denotes the gravity anomaly observed by the marine gravimeter, and $g_{map}$ denotes the gravity anomaly interpolated from a reference map according to the INS-indicated locations. The state noise $\left\{W_{k}\right\}$ and the measurement noise $\left\{V_{k}\right\}$ are unrelated zero-mean white-noise sequences. Further, q is the system noise and r is the measurement noise.

(2) $$E[{\left(W_{k}\right)}^{2}]=q,\;E[{\left(V_{k}\right)}^{2}]=r,$$

After Kalman filtering with multiple groups of filters in the confidence area, an estimated gravity anomaly $X_{k/k-1}$ was obtained for each group of filter. Then, each corresponding matched location was calculated. According to the Heli/SITAN algorithm [28], the degree of fit was best reflected by the difference $\delta_{k}$ between the measured and the estimated value. The residual $\delta_{k}$ could be expressed as dollows:

(3) $$\delta_{k}=Z_{k}-X_{k/k-1},$$

The smoothed weighted residual square (SWRS) that reflects the effect of multiple filtering was constructed on the basis of the residual. Then, the location corresponding to the filter with the minimum SWRS was the optimal one. Here, α denotes a smoothed weighted factor (0<α<1.0). $P_{k/k-1}=P_{k-1}+q$ is the estimated variance during the implementation of the Kalman filtering model, as shown by Equations (4) and (5).

(4) $$WRS_{k}={\left(\delta_{k}\right)}^{2}/(P_{k/k-1}+r),$$

(5) $$SWRS_{k}=\alpha WRS_{k}+(1-\alpha )SWRS_{k-1},$$

Upon the Kalman filtering of the multiple sets of filters in the confidence area, we obtained the estimated location for each set of filters. According to Equation (5), the smaller the SWRS value is, the better is the matching effect. Among the positions of the filters with different SWRS values in the confidence area, the position corresponding to the filter with the smallest SWRS value was the optimal matching position. The reliability of this optimal matching position was judged using the following criterion:

(6) $$H=\frac{SWRS_{\min}^{*}-SWRS_{\min}}{SWRS_{\min}}>H_{t},$$

where $SWRS_{\min}$ denotes the smallest value of all of the filters in the entire confidence region, $SWRS_{\min}^{*}$ indicates the smallest value of SWRS beyond a certain range with $SWRS_{\min}$ as the center, H represents the judgment criterion parameter, and $H_{t}$ refers to the threshold. A large value of H indicates significant differences between the $SWRS_{\min}$ and the $SWRS_{\min}^{*}$, and the larger the value is, the more prominent are the characteristics of the location corresponding to $SWRS_{\min}$. Therefore, the filter with $SWRS_{\min}$ is regarded as the optimal estimation filter, and the optimal matching location corresponding to $SWRS_{\min}$ is considered the effective location.

Considering the change in the marine gravity anomaly with distance, we found that the effective matching points had a typical spacing of 1–2 grids. The value function obtained using Equation (5) was the minimum, and the optimal location of the underwater carrier was localized on the gravity anomaly reference map. The matching location accuracy was considerably affected by the reference map resolution. In theory, the optimal matched location could be narrowed to 1 or even 1/2 of the gravity anomaly grid. In fact, when the gravity anomalies of 1 grid change were so small that they were overlaid by the noise of the gravity reference map error and of the gravimeter observations, we required 2–3 grids or a greater distance to ensure that the variation of the gravity anomaly met the requirement of high-precision matching and location determination. In the SITAN algorithm, the primary role of INS was to define a confidence search area of the real location. The matched location was not substantially affected by the INS drift as long as the real location was in its confidence area. Thus, with the GPS location as the benchmark, it performed two roles, i.e., (1) simulating the INS-indicated location on the basis of the inertial navigation accuracy and replacing INS before the matching location was begun; and (2), checking the matching location accuracy with IGNS after location matching was accomplished.

### 2.2. Variation Characteristics of Gravity Anomaly along the Route

The location accuracy of IGNS was mainly related to three factors, variation characteristics of the gravity anomaly reference map, resolution and accuracy of this map, and observational accuracy of the marine gravimeter. Therefore, we had to pre-analyze the characteristics of the gravity anomaly along the route [29,30].

**Figure 2:**
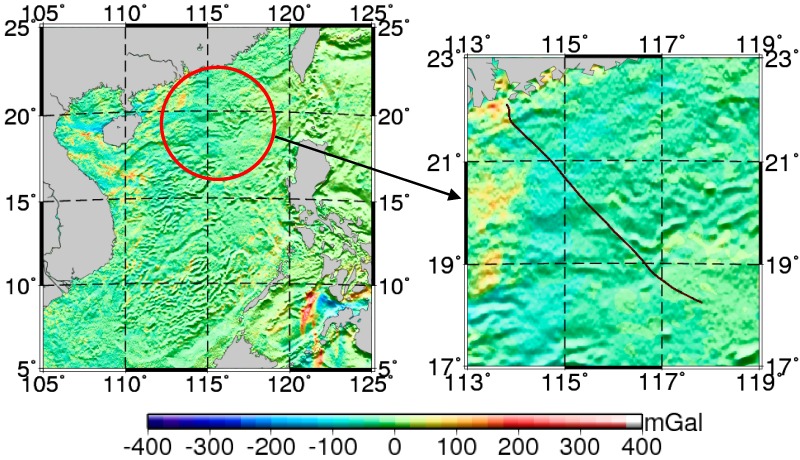
Ship-measured gravity areas and tracks.

In this work, the data used in the ocean experiment were measured in the South China Sea during an exploration mission. These contained gravity data were measured using a Lacoste marine gravimeter and GPS location information along the route. Figure 2 shows the location of the ship-measured gravity area and its corresponding route. The total length of the route was ~340 n miles. The global marine gravity anomaly model grav.img.24.1 [31] was used for the marine gravity reference map. The reference map was constructed by the Scripps Institution of Oceanography (La Jolla, CA, USA) and had a grid resolution of 1′ × 1′. Compared to the ship-measured gravity, its overall accuracy was 3–8 mGal [32,33].

The location accuracy of gravity matching was closely related to the variation characteristics of the route gravity anomaly and the degree of fit between the reference map and the gravimeter observation data. For the measured gravity data $\Delta g_{i}$ (see $g_{meas}$ in Equation (1)) from the ship route, the mean was set to $\bar{\Delta g}$ and dispersion to D, as shown in Equations (7) and (8).

(7) $$\bar{\Delta g}=\frac{1}{n}\sum_{i=1}^{n}\Delta g_{i},$$

**Figure 3:**
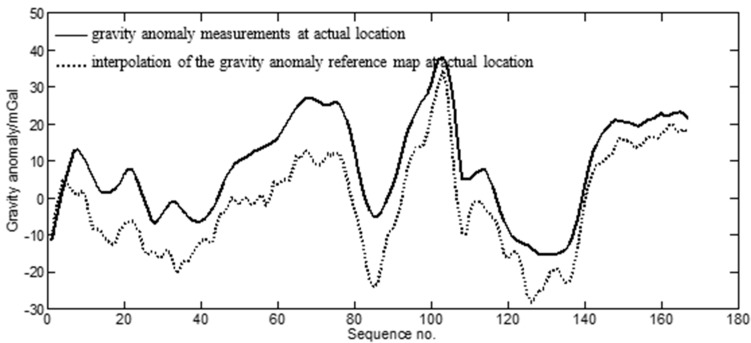
Comparison between gravimeter-measured data along the route and gravity anomaly interpolation at corresponding location of the reference map.

(8) D=[1n∑i=11(Δgi−Δg¯)2]12,

Table 1 shows the preliminary results of the statistical characteristics of the measured gravity anomalies $\Delta g_{i}$. $\bar{\Delta g}$ was 8.5 mGal and dispersion D was 13.3 mGal. The D value of the measured gravity anomalies indicated the fluctuation (or magnitude of variation) of the gravity anomaly along the route. The greater the dispersion was, the more prominent were the gravity characteristics. The location of the expedition ship was given by GPS. Correspondingly, gravity anomalies (see $g_{map}$ in Equation (1)) along the route were obtained by an interpolation of the gravity anomaly reference map according to the GPS location. The gravimeter-measured data along the route and those of the above interpolation were qualitatively compared, as shown in Figure 3.

The sequence of gravity difference between the gravimeter-measured gravity anomaly along the route and the interpolation of the gravity anomaly reference map at the corresponding locations was defined by $\sigma_{i}$ (i=1,2,⋯,n). The degree of fit between the two was F, as shown by the following:

(9) F=(1n∑i=1nδi2)12,

The statistical characteristics of the aforementioned difference are shown in Table 2. According to this table, there was a 10.3-mGal systematic difference and a 4.3-mGal standard deviation between the measured gravity anomaly and the interpolated gravity anomaly at the corresponding locations on the reference map. F represents the degree of fit between the measured gravity anomaly and the gravity anomaly at the corresponding location of the reference map. F=11.18 mGal, including the 10.3-mGal systematic difference and the 4.3-mGal standard deviation. As shown in Figure 3 and Table 2, the overall trend and characteristics were reasonably consistent between the two datasets, despite a substantial systematic difference.

## 3. Results of Matching Location on the Basis of Ocean Experiment and Simulation

The assessment of the performance and the location accuracy of IGNS was critical. A matching location determination outcome was evaluated experimentally under actual ocean conditions or compared with a simulation, thereby identifying the effects on the matching location accuracy.

### 3.1. Results of Matching Location with Ocean Experiment

**Figure 4:**
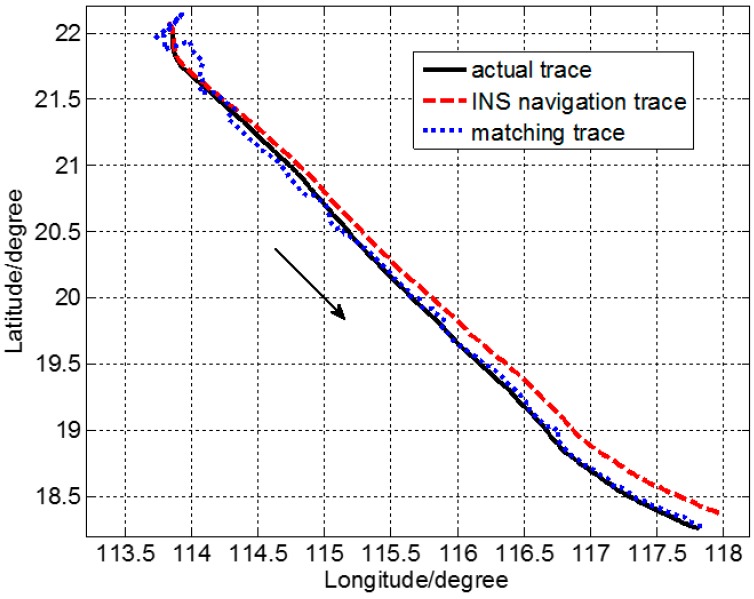
Matching and location trace during the ocean experiment.

Data were collected using a shipboard GPS instrument and a marine gravimeter with the Lacoste marine gravimeter. Using the high-precision GPS location data and the high-precision gravity data observed along the route, we used the SITAN algorithm for the ocean experiment of IGNS. The GPS location information was taken as the reference to check the system location accuracy. However, it was not used in the SITAN algorithm. In addition, because INS devices were not carried in the ocean experiment, INS location information was simulated according to the GPS location. The error of the INS initial location was 0.1 n miles, the gyroscopic drift rate was 0.005°/h, and the accelerometer error was 5 × 10^−^^6^ *g* (1 *g =* 9.8 m/s^2^). Then, the INS location was obtained by simulation under the aforementioned conditions. Corresponding to Figure 1, *d* was set to 16 n miles to ensure that the real location was in the confidence region centered on the INS-indicated location. Considering the accuracy of the gravity anomaly reference map and the measurement error of the marine gravimeter, *q* = 1.0 mGal^2^ and *r* = 10.0 mGal^2^ in Equation (2). A matching location result was calculated every 2.0 n miles using SITAN. There were 167 matching points in all. The computational time of each matching point was approximately 5 s with an 8-GB internal storage. The matching location track is shown in Figure 4. The real ship-measured track (i.e., GPS location) is marked by ——, the simulated track from the INS by – – –, and the gravity matching-aided navigation location by ……. The direction that the carrier traveled is marked with an arrow.

SITAN was essentially based on a Kalman filtering algorithm. Because of the considerable error in the estimated initial state and initial variance in the Kalman filtering equation, large differences were observed between the estimated initial value of Kalman filtering and the real initial value. As the number of iterations increased, the variance declined and the prediction accuracy increased. Nevertheless, the improvement was only substantial in the first few steps. After several iterations, the filtering gain stabilized, and the tracking object was generally well predicted. As shown in Figure 4, initially large matching and location errors were observed, but all of them converged quickly and we managed to track the target location.

**Figure 5:**
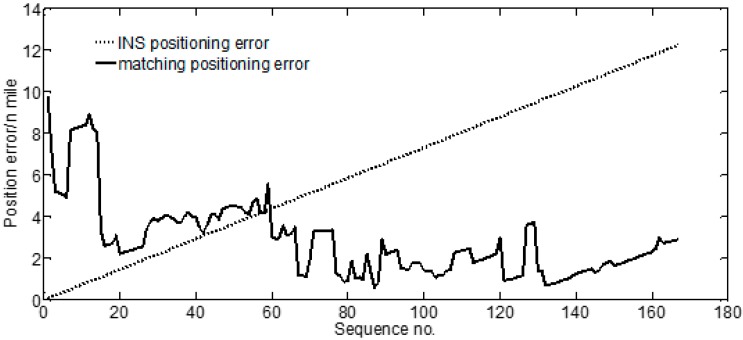
Comparison between error of the INS-indicated location and the error of the matched location.

Corresponding to Figure 4, Figure 5 shows location error of each matching point. The location error of the INS was observed to accumulate over time and reach ~12 n miles. The location error of IGNS changed slightly within a certain range. The matching location effects of this system are qualitatively reflected in Figure 4 and Figure 5. The matching location accuracy of the route was statistically analyzed for further quantitative research. The location error $\upsilon^{i}$ (i=1,2,⋯,n) of each matching point was defined as shown in Equation (10). Here, n denotes the total number of matching points.

(10) $$\upsilon^{i}=\sqrt{{\left(\lambda_{gps}^{i}-\lambda_{matching}^{i}\right)}^{2}+{\left(\varphi_{gps}^{i}-\varphi_{matching}^{i}\right)}^{2}}$$

where ($\lambda_{gps}^{i},\varphi_{gps}^{i}$) denotes the GPS-identified location and ($\lambda_{matching}^{i},\varphi_{matching}^{i}$) represents the location identified through matching by IGNS. The mean, standard deviation and root mean square of $\upsilon^{i}$ were statistically analyzed; the corresponding results are shown in Table 3. The overall matching location accuracy was observed to reach 2.83 n mile.

### 3.2. Results of Matching Location with Simulation

Given the complexity of ocean experiments, few studies have included the actual ocean experiments of IGNS. However, extensive simulation studies have been conducted, but their results have not been compared with experimental results. In the present work, we simulated the matching location of the abovementioned system under different noise conditions on the same route, and analyzed the effect of these conditions on the matching location outcome in combination with an actual ocean experiment.

The simulation parameters and *d* of the INS-indicated location and the system noise and observation noise in Kalman filtering were the same as those reported in Section 3.2. In contrast to the ocean experiment, the observed data of the marine gravity anomaly from the marine gravimeter were obtained from simulation. In the absence of actual marine gravity anomaly data from the marine gravimeter, the interpolation value $\Delta g^{\prime }$ on the gravity anomaly reference map corresponding to the GPS location was taken as the reference. Bilinear interpolation was used to calculate $\Delta g^{\prime }$. In IGNS, we mainly considered relative matching between the gravimeter observation data and interpolation of the gravity anomaly reference map, whereas in the simulation, the observation error of the gravimeter and the error of the gravity anomaly reference map were considered as a whole. In light of the current accuracy of the marine gravimeter and the gravity anomaly reference map, the mean was defined as u and the variance as $\sigma^{2}$. Then, the appropriate preference for u and $\sigma^{2}$ was set within a certain range. Bilinear interpolation was used to calculate the $\Delta g^{\prime }$ of the reference map at an actual location on the route. The random seed number *n* was consistent with the number of measurement points in the ocean experiment discussed in Section 3.2. Then, the gravity anomaly noise with a two-dimensional normal distribution $\Delta v\sim N(u,\sigma^{2})$ was obtained. Simulation observations ${g^{\prime }}_{meas}$ were obtained by adding Δv and $\Delta g^{\prime }$. That is, ${g^{\prime }}_{meas}=\Delta v+\Delta g^{\prime }$.

During the location matching of IGNS, the core step was the matching between the gravity anomaly observations and the gravity anomaly reference map. Their difference was mainly reflected by Δv. To analyze the influence of u and $\sigma^{2}$ on the matching location effects, simulations were run for the cases of fixed u and $\sigma^{2}$, respectively. According to Equation (9), the matching and location accuracy of simulation with a mean u of 0 mGal and variance $\sigma^{2}$ of 1–25 mGal^2^ are shown in Table 4. Further, results with a variance $\sigma^{2}$ of 9 mGal^2^ and mean u of 0–4 mGal are shown in Table 5.

Table 4 shows that at a constant mean value u and with a gradual increase in the noise variance $\sigma^{2}$ from 1 to 25 mGal^2^, the root mean square, a key indicator of location accuracy, gradually increased from 1.2 to 4.0 n miles. Table 5 shows that at a constant noise variance $\sigma^{2}$ and mean u 0–4 mGal, the location accuracy remains relatively stable at 2.03–2.58 n miles without any major changes. Given the same u and $\sigma^{2}$, as the random seed of the normal distribution changes in every simulation and despite the fact that the simulated noise is generally subject to a two-dimensional normal distribution $\Delta v\sim N(u,\sigma^{2})$, the results of each simulation varied slightly. This might result in a minor fluctuation in the simulated matching location results. As shown in Table 5, the location error with u = 3 mGal was only 0.25 n miles less than that for u = 0 mGal. Thus, the results presented in Table 5 were considered to be consistent within a reasonable error range.

According to Table 4 and Table 5, of all the difference characteristics between the observed gravity anomaly and from the interpolation of the reference map, the mean value u had no major influence on the matching location accuracy, whereas the variance of noise $\sigma^{2}$ mainly affected the matching results. The mean value u represented the systematic difference and the variance $\sigma^{2}$ directly reflected the characteristic difference. Table 4 shows not only qualitatively that the greater the variance was, the more important was the characteristic difference and the lower was the accuracy, but also quantitatively that when the noise variance increased from 1 to 25 mGal^2^, the accuracy decreased from 1.2 to 4.01 n miles.

**Figure 6:**
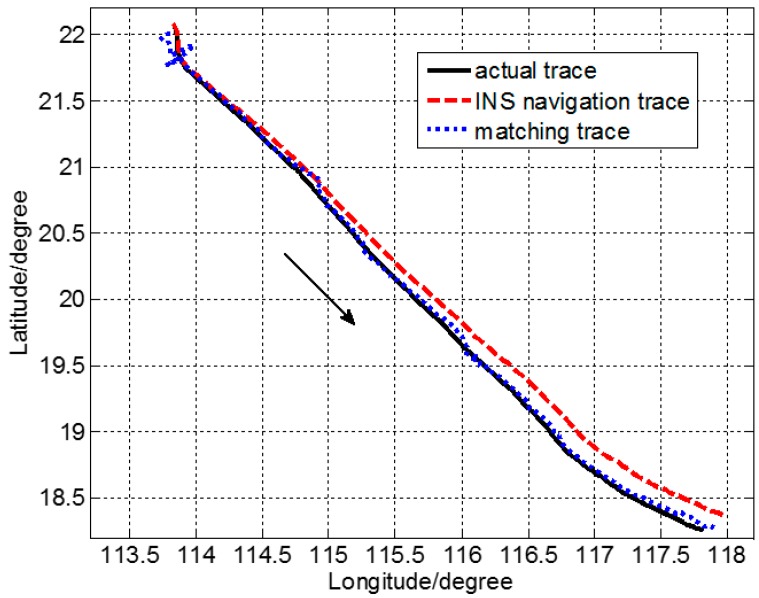
Matching location trace in the simulation under noise condition *u* = 1 mGal and *σ*^2^ = 9 mGal^2^.

In all, 10 simulations were conducted under different noise conditions, as shown in Table 4 and Table 5. As an example, the results *u* = 1 mGal and *σ*^2^ = 9 mGal^2^ are shown in Figure 6, which reflect the location outcome qualitatively and macroscopically.

According to Table 2, a comparison between the ship-measured gravity anomaly obtained from the actual ocean experiment and the gravity interpolation at the corresponding locations of the reference map showed that the mean was 10.31 mGal and the standard deviation was 4.32 mGal. Based on the statistical characteristics of the difference between the ship-measured gravity and the gravity at the corresponding locations of the reference map, a simulation was carried out using roughly the same noise condition (*u* = 10.0 mGal; *σ* = 4.0 mGal). Table 6 shows the statistical results of the matching location accuracy. Table 3 shows that the location accuracy of the actual ocean experiment was 2.83 n miles. Table 6 shows that the simulation accuracy under the same noise condition on the same route was 2.96 n miles. Given the resolution of the reference map (1.0 × 1.0 n miles), the location error of 0.5 n miles was within a reasonable range; therefore, the results of the ocean experiment were in agreement with those from the simulation within a reasonable error range.

## 4. Discussion and Conclusions

Based on the existing computer simulations of gravity matching-aided navigation, real-time ship-measured gravity data and high-precision GPS location data were used to conduct a gravity matching-aided navigation experiment on a recent measurement route in the South China Sea. The results were then compared with those obtained from a simulation under different noise conditions. This showed that the systematic difference between the gravity anomaly observations and the gravity reference map had no major influence on the matching location accuracy, whereas the noise variance considerably affected the results. With an increase in this variance, the matching location error gradually increased. Its quantitative effect was presented. The consistency of the accuracy of the ocean experiment and the simulation demonstrated the effectiveness of the proposed simulation method and the rationality of the noise influence on the location accuracy. In addition, we calculated the location accuracy that can be attained by IGNS given current hardware and software.

IGNS has strong autonomy and effective concealment, so it can suppress the accumulation of the navigation error and is thus suitable for long-distance, long-period navigation. However, the system is constrained by gravitational field characteristics in various regions; its effect is not ideal where a change in the gravitational field is not substantial, such as the localized areas of the East China Sea and the Western Pacific [34,35]. Limited by the current resolution (1.0 × 1.0 n miles) and accuracy of the reference map (3–8 mGal), the theoretical location accuracy can reach 0.5 grid (i.e., 0.5 n miles). However, because the gravity anomaly varies with distance, the accuracy is 1.0–3.0 grids in most cases; thus, it can be used for long-distance autonomous navigation with a location accuracy of 1.0–3.0 n miles. With the continuous development of satellite altimetry technology, a new generation of altimetric satellites is expected to be launched in succession. With the integration of new satellite data into the current gravity field model, in the future, the resolution and precision of the reference map of the ocean gravitational field will be further enhanced.

Extensive simulation research has been conducted on IGNS, but the results have never been compared with an actual ocean experiment. In simulation, a high-precision gravity reference map with strongly varying gravity can be simulated, and noise can be controlled to an extremely low level. In this case, satisfactory matching location results can be achieved using IGNS. Given the existing marine gravimeters and marine gravity reference maps, a comparative analysis of the ocean experiment and the simulation conducted in this study is very useful for evaluating the feasibility and navigation performance of the proposed navigation system. However, the area covered by the route in this study was limited. The performance of the navigation system should be carefully investigated in different critical scenarios, so as to fully assess the accuracy and reliability of the gravity-aided navigation system and to further explore the IGNS theory and the related simulation system.

Experimental results (B. Allotta, et al.) exhibited a satisfactory localization accuracy for both EKF and UKF, the latter being more accurate than the former. The achieved results can serve as a reference for future online tests with the vehicle (both in simulation and during experimental campaigns) [36]. In view of the advantages and disadvantages of the different matching algorithms currently available, theories that integrate various matching algorithms will attract considerable research attention. To further improve the location accuracy of underwater submersibles during long-distance and long-period sailings, navigation information collected via observation sensors from various information sources (such as inertial navigation, gravity, hydroacoustics, GNSS, and submarine terrain) can be combined. This approach can be used for information theories with shared time and spatial frameworks determined by underwater submersibles, thereby realizing long-distance, long-period, and real-time dynamic location of underwater submersibles with considerable precision [37,38].

## Figures and Tables

**Table 1:** Statistical characteristics of the gravity anomaly observations along the route (unit: mGal).

Min.	Max.	Mean $\bar{\Delta g}$	Dispersion D
−15.40	38.14	8.50	13.30

**Table 2:** Statistical characteristics of the difference between the observed gravity anomaly along the route and the gravity at the corresponding location of the reference map (unit: mGal).

Min.	Max.	Mean	STD	Degree of Fit F
−1.28	19.77	10.31	4.32	11.18

**Table 3:** Statistical results of matching and location errors (unit: n mile).

Min.	Max.	Mean	STD	RMS
0.58	9.75	2.49	1.33	2.83

**Table 4:** Matching location accuracy in the simulation with mean = 0 mGal and varying variance.

Simulation of Noise Conditions	Mean	STD	RMS
u=0, $\sigma^{2}=1$	1.05	0.59	1.20
u=0, $\sigma^{2}=4$	1.57	0.84	1.78
u=0, $\sigma^{2}=9$	1.89	1.30	2.30
u=0, $\sigma^{2}=16$	2.18	2.04	2.99
u=0, $\sigma^{2}=25$	3.13	2.49	4.01

**Table 5:** Matching location accuracy in the simulation with variance = 9 mGal^2^ and varying mean values.

Simulation of Noise Conditions	Mean	STD	RMS
u=0, $\sigma^{2}=9$	1.89	1.30	2.30
u=1, $\sigma^{2}=9$	1.88	1.24	2.25
u=2, $\sigma^{2}=9$	1.57	1.29	2.03
u=3, $\sigma^{2}=9$	1.62	1.26	2.05
u=4, $\sigma^{2}=9$	2.01	1.60	2.58

**Table 6:** Statistical results of matching location accuracy in the simulation.

Simulation of Noise Conditions	Mean	STD	RMS
u=10, $\sigma^{2}=16$	2.21	1.96	2.96
